# Effects of Intravenous Infusion of Lidocaine on Short-Term Outcomes and Survival in Patients Undergoing Surgery for Ovarian Cancer: A Retrospective Propensity Score Matching Study

**DOI:** 10.3389/fonc.2021.689832

**Published:** 2022-01-06

**Authors:** Hao Zhang, Jiahui Gu, Mengdi Qu, Zhirong Sun, Qihong Huang, Juan P. Cata, Wankun Chen, Changhong Miao

**Affiliations:** ^1^ Department of Anesthesiology, Zhongshan Hospital, Fudan University, Shanghai, China; ^2^ Department of Anesthesiology, Fudan University Shanghai Cancer Centre, Shanghai, China; ^3^ Department of Oncology, Shanghai Medical College, Fudan University, Shanghai, China; ^4^ Cancer Center, Zhongshan Hospital, Fudan University, Shanghai, China; ^5^ Shanghai Respiratory Research Institute, Shanghai, China; ^6^ Institute of Clinical Sciences, Zhongshan Hospital, Fudan University, Shanghai, China; ^7^ Department of Anesthesiology and Perioperative Medicine, The University of Texas MD Anderson Cancer Centre, Houston, TX, United States; ^8^ Anesthesiology and Surgical Oncology Research Group, Houston, TX, United States

**Keywords:** lidocaine infusion, SCN9A, ovarian cancer, overall survival, disease-free survival

## Abstract

**Background:**

Intravenous lidocaine has been shown to reduce opioid consumption and is associated with favourable outcomes after surgery. In this study, we explored whether intraoperative lidocaine reduces intraoperative opioid use and length of stay (LOS) and improves long-term survival after primary debulking surgery for ovarian cancer and explored the correlation between SCN9A expression and ovarian cancer prognosis.

**Methods:**

This retrospective study included patients who underwent primary debulking surgery(PDS) for ovarian cancer from January 2015 to December 2018. The patients were divided into non-lidocaine and lidocaine [bolus injection of 1.5 mg/kg lidocaine at the induction of anaesthesia followed by a continuous infusion of 2 mg/(kg∙h) intraoperatively] groups. Intraoperative opioid consumption, the verbal numeric rating scale (VNRS) at rest and LOS were recorded. Propensity score matching was used to minimize bias, and disease-free survival (DFS) and overall survival (OS) were compared between the two groups.

**Results:**

After propensity score matching(PSM), the demographics were not significantly different between the groups. The intraoperative sufentanil consumption in the lidocaine group was significantly lower than that in the non-lidocaine group (Mean: 35.6 μg vs. 43.2 μg, P=0.035). LOS was similar between the groups (12.0 days vs. 12.4 days, P=0.386). There was a significant difference in DFS between the groups (32.3% vs. 21.6%, P=0.015), and OS rates were significantly higher in the lidocaine group than in the non-lidocaine group (35.2% vs. 25.6%, P=0.042). Multivariate analysis indicated that intraoperative lidocaine infusion was associated with prolonged OS and DFS.

**Conclusion:**

Intraoperative intravenous lidocaine infusion appears to be associated with improved OS and DFS in patients undergoing primary debulking surgery for ovarian cancer. Our study has the limitations of a retrospective review. Hence, our results should be confirmed by a prospective randomized controlled trial.

## Introduction

Ovarian cancer ranks third among the most common gynaecologic tumours and is the eighth leading cause of cancer-related death in women in developing countries (1). Despite significant progress in the treatment of ovarian cancer, the 5-year survival rate remains lower than 50% (2). This worrisome statistic highlights the urgent need to find therapies that can contribute to reducing ovarian cancer progression and thus improve patient survival. Preclinical and clinical studies suggest that anaesthetic agents may influence cancer biology and outcomes (3). Anaesthetics and inadequate pain control might also be associated with the risk of metastatic recurrence after ovarian cancer surgery (4–6). Therefore, current research is focused on improving the survival of women with ovarian cancer by optimizing perioperative management.

Lidocaine is a widely used amide local anaesthetic that can reduce general anaesthetic use, minimize opioid consumption and provide adequate analgesia when given systemically during oncological surgery (7). In addition, lidocaine has shown promising anticancer properties. Different mechanisms have been described as being responsible for the antimetastatic effects of lidocaine, including TRPV6 receptor inhibition (8), reduced epidermal growth factor activity (9), and time- and dose-dependent deoxyribonucleic acid demethylation in different cancer cell lines (10). Moreover, voltage-gated sodium channels (VGSCs) are expressed in cancer cells, controlling important mechanisms of the metastatic process (11). In support of these preclinical findings, the results of our previous study indicate that perioperative intravenous infusion of lidocaine is associated with longer overall survival (OS) in patients with pancreatic cancer (12).

It is unclear whether intravenous lidocaine infusion during ovarian cancer surgery is associated with any improvement in surgical recovery and long-term oncologic outcomes. Thus, the aim of this retrospective study was to evaluate the association between intravenous infusion of lidocaine during ovarian cancer surgery and intraoperative sufentanil consumption, postoperative analgesia scores, and long-term patient survival outcomes. Since lidocaine acts on sodium voltage channels, we also explored the association between *SCN9A* gene expression and long-term prognosis in ovarian cancer patients.

## Methods

### Patients Enrolment

This study was approved by the Ethics Committee of Fudan University (no. 20200206). From January 2015 to December 2018, patients undergoing primary debulking surgery (Ro resection) for ovarian cancer were enrolled as a retrospective cohort. The inclusion criteria were as follows: (a) 18 years or older; (b) received combined general-epidural anaesthesia and (c) complete clinicopathological and follow-up data. Patients were excluded if they (a) underwent emergency surgery, (b) had any history of another malignant tumour, (c) died within 30 days of surgery from postoperative complications or (d) were lost to follow-up.

We collected demographic information [age, body mass index and American Society of Anaesthesiologists (ASA) physical status], Charlson Comorbidity Index (CCI), pathological details (tumour size, histologic diagnosis, FIGO stage and tumour differentiation), surgical details (surgical complexity, estimated blood loss volumes and operative time), preoperative CA125 levels and postoperative chemotherapy. We obtained one to five years of follow-up (every three months for the 1st and 2nd years and every six months for the 3rd year) from medical records and telephone contacts.

### Endpoints

The primary endpoints of interest were disease-free survival (DFS) and overall survival (OS). OS was defined as the period from the patient’s date of surgery to the time of death or last follow-up. DFS was defined as the interval between the date of surgery and the date of tumour recurrence or December 31, 2019. Follow-up was continued until December 31, 2019, or until the patient died. The secondary endpoints included intraoperative sufentanil consumption, the verbal numeric rating scale (VNRS) at rest and the length of postoperative hospital stay.

### Exposure Variable

We sought to determine the effect of lidocaine on short- and long-term outcomes after primary debulking surgery. Patients in the lidocaine group received an initial bolus of lidocaine (1.5 mg/kg) at the induction of general anaesthesia, followed by a continuous infusion of 2 mg/(kg∙h) intraoperatively that was stopped at the end of surgery. In the non-lidocaine group, the patients in non-lidocaine group receive continuous bolus injection of 1.5 mg/kg saline at the induction of anaesthesia followed by a continuous infusion of 2 mg/(kg∙h) intraoperatively. Epinephrine was not used combined with lidocaine infusion. The decision to initiate lidocaine therapy was made by the attending anaesthesiologist assigned to the case and based on clinical judgement.

### Anaesthesia Care

Upon entering the operating room, all patients were monitored according to ASA monitoring standards. In all patients, general anaesthesia was induced with propofol (target-controlled infusion, effect-site concentration: 3.0-4.0 µg/ml), sufentanil (0.3-0.5 µg/kg), and rocuronium (0.6 mg/kg). The patients were then endotracheally intubated, and general anaesthesia was maintained with 2.0-3.0% sevoflurane in an oxygen/air mixture. Repeated bolus injections of sufentanil and rocuronium were given as necessary throughout the operation. All patients received 5ml of 0.375% ropivacaine after the induction of general anaesthesia, plus 4ml of ropivacaine every 50 minutes until the end of surgery. All patients in the study received epidural analgesia with an infusion of 0.375% ropivacaine *via* an epidural catheter placed at the mid-thoracic level (T12-S1). At the end of the operation, all patients in both groups received a patient-controlled epidural analgesia (PCEA) pump (0.1% ropivacaine and 0.5 μg/ml sufentanil, background: 2-3 ml/h, bolus: 3-4 ml, lockout time: 15 min) for 48 h.

### *SCN9A* mRNA Expression Analysis

The Kaplan-Meier (KM) Plotter database contains reliable *SCN9A* mRNA expression for 675 patients (13). Patients with *SCN9A* mRNA tumour information in the database were identified from Cancer Biomedical Informatics Grid (http://cabig.cancer.gov/), Gene Expression Omnibus (http://www.ncbi.nlm.nih.gov/geo/) and The Cancer Genome Atlas (http://cancergenome.nih.gov) cancer datasets. We analysed progression-free survival and OS as provided by the KM plotter database.

### Statistical Analysis

Patient demographics, disease status, intraoperative variables and outcomes were summarized through descriptive statistics. Categorical data were analysed with the chi-square test, and the results are expressed as N (%); continuous data are expressed as the mean ± standard deviation (SD), and two independent samples were analysed with the t-test. Fisher’s exact test or the chi-square test was used to evaluate associations between categorical variables. T-tests or Mann-Whitney U tests were employed to compare continuous variables between patient groups. The Kaplan-Meier method was applied to calculate OS and DFS. To assess the prognostic value of *SCN9A*, each percentile (of expression) between the lower and upper quartiles and the median threshold was used as the final cut-off in univariable Cox regression analysis. To evaluate the impact of lidocaine on survival, Cox proportional hazards regression models were used to compare risk factors between the different groups by using univariate models. Variables that were significant in the univariate analysis were entered into a multivariate model using the forward conditional method, which was used to fit the multivariate model. We performed propensity score matching analysis to adjust for selection bias in the observational study. The following variables were entered in our propensity model: age, ASA grade, Charlson comorbidity, FIGO stage, histologic diagnosis, tumour differentiation, residual disease, and adjuvant chemotherapy. Statistical analyses were performed with SPSS 17.0 (SPSS Inc., Chicago, IL, U.S.A.), and a P-value <0.05 was considered statistically significant.

## Results

A total of 702 patients who underwent primary debulking surgery for ovarian cancer were enrolled in this study, with 376 in the non-lidocaine group and the remaining 326 in the lidocaine infusion group. After propensity score matching, 302 patients remained in the non-lidocaine infusion group and 302 in the lidocaine infusion group. The patients’ demographics, including age, ASA physical status, operative variables, and FIGO stage, were similar between the groups (**Table 1**).

**Table 1:** Patient and treatment characteristics for both groups.

Variable	Original cohort		*P*	Matched cohort		*P*	Standard difference (%)
	Non-lidocaine group (n=376)	Lidocaine group (n=326)		Non-lidocaine group (n=302)	Lidocaine group (n=302)		
**Age (years)**	53.6±10.6	54.2±11.2	0.460	53.2±10.2	53.4±10.6	0.813	3.26
**BMI (kg/m2)**	27.6±6.5	28.6±6.2	0.382	27.3±6.2	27.4±6.3	0.844	–
**ASA (n, %)**			0.893			0.927	4.12
I-II	283 (75.2%)	243 (74.6%)		242 (80.1%)	241 (79.8%)		
III-IV	93 (24.8%)	83 (25.4%)		80 (19.9%)	81 (20.2%)		
**Patients enrolled**			<0.001			0.998	–
2015	93 (24.7%)	76 (23.2%)		73 (24.1%)	71 (23.5%)		
2016	92 (24.5%)	78 (23.8%)		72 (23.8%)	73 (24.2%)		
2017	95 (25.3%)	80 (24.5%)		81 (26.8%)	81 (26.9%)		
2018	96 (25.5%)	92 (28.5%)		76 (25.3%)	77 (25.4%)		
**CCI (n, %)**			0.942			0.964	5.26
0	62 (16.5%)	55 (16.9%)		59 (19.5%)	58 (19.3%)		
1	172 (45.7%)	152 (46.5%)		159 (52.6%)	157 (51.9%)		
≧2	142 (37.8%)	119 (36.6%)		84 (27.9%)	87 (28.8%)		
**Histologic diagnosis**			0.903			0.930	4.25
Serous histology	236 (62.8%)	207 (63.5%)		210 (69.5%)	208 (68.9%)		
Non-serous histology	140 (37.2%)	119 (36.5%)		92 (30.5%)	94 (31.1%)		–
**Tumor size**			0.801			0.932	
>5	218 (57.9%)	185 (56.8%)		195 (64.6%)	194 (64.2%)		
<5	158 (42.1%)	141 (43.2%)		107 (35.4%)	108 (35.8%)		
**FIGO stage (n, %)**			0.950			0.995	3.28
I	32 (8.5%)	30 (9.3%)		26 (8.6%)	25 (8.4%)		
II	43 (11.4%)	35 (10.7%)		35 (11.6%)	36 (11.8%)		
III	168 (44.7%)	141 (43.5%)		159 (52.6%)	157 (51.9%)		
IV	133 (35.4%)	120 (36.5%)		82 (27.2%)	84 (28.2%)		
**Tumor differentiation**			0.991			0.965	6.25
Well	36 (9.6%)	32 (9.8%)		29 (9.6%)	28 (9.3%)		
Moderate	215 (57.2%)	185 (56.9%)		165 (54.6%)	163 (53.9%)		
Poor	125 (33.2%)	109 (33.3%)		108 (35.8%)	111 (36.8%)		
**Residual disease**			0.919			0.878	4.65
No visible disease	186 (49.5%)	159 (48.7%)		156 (51.6%)	154 (51.0%)		
<1cm residual disease	132 (35.1%)	119 (36.4%)		113 (37.4%)	111 (36.9%)		
>1cm residual disease	58 (15.4%)	48 (14.9%)		33 (11.0%)	37 (12.1%)		
**Surgical complexity**			0.829			0.986	–
Low	62 (16.5%)	56 (17.2%)		43 (14.2%)	44 (14.5%)		
Intermediate	200 (53.2%)	178 (54.6%)		162 (53.6%)	160 (52.9%)		
High	114 (30.3%)	92 (28.2%)		97 (32.2%)	98 (32.6%)		
**Operation time (min)**	213±63	209±59	0.388	202±61	206±62	0.424	–
**Ascites (ml)**			0.929			0.961	–
<200	62 (16.5%)	56 (17.3%)		45 (14.9%)	44 (14.6%)		
>200	53 (14.1%)	45 (13.9%)		43 (14.2%)	44 (14.5%)		
**Estimated blood loss (n, %)**			0.730			0.953	–
≤ 400 ml	219 (58.2%)	195 (59.7%)		195 (64.6%)	194 (64.2%)		
> 400 ml	157 (41.8%)	131 (40.3%)		107 (35.4%)	108 (35.8%)		
**Blood transfusion**			0.912			0.934	–
No	235 (62.5%)	206 (63.2%)		185 (61.3%)	183 (60.5%)		
Yes	141 (37.5%)	120 (36.8%)		117 (38.7%)	119 (39.5%)		
**Pre CA125 (U/ml)**	635 (182-1126)	663 (172-1047)	0.462	626 (194-985)	610 (202-798)	0.541	–
**Postop-Chemotherapy (n, %)**			0.902			0.931	6.25
No	123 (32.7%)	109 (33.5%)		100 (33.1%)	98 (32.5%)		
Yes	253 (67.3%)	217 (66.5%)		202 (66.9%)	204 (67.5%)		
**Postop-Chemotherapy across year (n, %)**			0.981			0.937	–
2015	62 (16.5%)	51 (15.8%)		53 (17.5%)	52 (17.3%)		
2016	64 (17.0%)	54 (16.7%)		50 (16.6%)	48 (15.8%)		
2017	68 (18.1%)	58 (17.8%)		53 (17.5%)	52 (17.2%)		
2018	59 (15.7%)	54 (16.2%)		46 (15.3%)	52 (17.2%)		

BMI, Body Mass Index; ASA, American Society of Anesthesiologists score; CCI, Charlson Comorbidity Index; FIGO, Federation International of Gynecology and Obstetrics.

### Primary Endpoint

**Figure 1:**
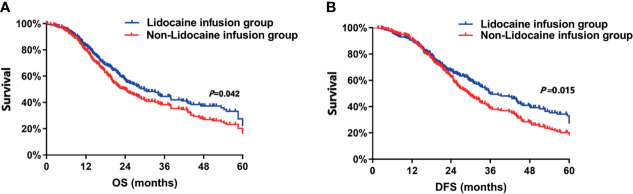
**(A)** Overall survival curves from the date of surgery according to the use of intraoperative intravenous lidocaine infusion. **(B)** Disease-free survival curves from the date of surgery according to the use of intraoperative intravenous lidocaine infusion. DFS, disease-free survival; OS, overall survival.

In this study, the median follow-up time for all patients was 46.8 months. The Kaplan–Meier survival curves for the lidocaine infusion and non-lidocaine infusion groups are displayed in **Figure 1**. The Kaplan-Meier curves for OS suggest that patients who were treated with lidocaine had a significant improvement in survival. Indeed, OS rates at 3 years and 5 years after surgery were significantly higher among the patients in the lidocaine infusion group than in the non-lidocaine infusion group [3-year OS: 45.2%, vs. 37.5%, P<0.001, and 5-year OS: 35.2% vs. 25.6%, P=0.011, respectively, **Figure 1A**]. In addition, univariate Cox regression analysis showed that age, ASA score, CCI, tumour differentiation, FIGO stage, residual disease, surgical complexity, ascites, intraoperative blood loss, adjuvant chemotherapy and lidocaine infusion were associated with OS (**Table 2**). According to multivariate analysis before propensity score matching, the following covariates were significantly associated with unfavourable OS: poor tumour differentiation (HR: 1.36, 95% CI: 1.02, 1.78, P=0.011), residual disease (HR: 1.28, 95%CI: 1.23, 1.58, P<0.001), and no adjuvant chemotherapy (HR: 1.56, 95% CI: 1.41, 1.62, P<0.001) (**Table 3**). Intravenous infusions of lidocaine were associated with prolonged OS (HR: 0.93, 95% CI: 0.82, 0.98, P=0.026). After propensity score matching, the association between lidocaine infusion and OS remained statistically significant (HR: 0.86, 95% CI: 0.62, 0.98, P=0.038). The following covariates were also statistically significant: tumour differentiation (HR: 1.26, 95% CI: 1.22, 1.73, P=0.011), residual disease (HR: 1.21, 95%CI: 1.02, 1.48, P=0.029), and no adjuvant chemotherapy (HR: 1.16, 95% CI: 1.12, 1.43, P=0.016) (**Table 3**).

**Table 2:** Univariate analysis of OS and DFS.

Variables	OS		DFS	
	HR (95% CI)	*P*-value	HR (95% CI)	*P*-value
**Age (years)**	1.06 (1.02-1.12)	0.025	1.12 (1.08-1.26)	0.013
**BMI (kg/m2)**	1.00 (0.95-1.05)	0.265	1.02 (1.00-1.16)	0.352
**ASA score (III-IV)**	1.48 (1.22-1.73)	0.016	1.23 (1.14-1.28)	0.036
**CCI (>2)**	1.26 (1.20-1.56)	0.035	1.32 (1.11-1.42)	0.019
**Histologic diagnosis (Non-serous histology)**	1.35 (1.16-1.65)	<0.001	1.42 (1.32-1.63)	<0.001
**Tumor differentiation (poor)**	1.46 (1.12-1.68)	0.035	1.46 (1.26-1.83)	0.042
**FIGO stage (III-IV)**	1.68 (1.15-2.15)	<0.001	1.59 (1.12-1.78)	<0.001
**Residual disease (>1cm)**	1.82 (1.45-2.16)	0.028	1.95 (1.26-2.06)	0.014
**Surgical complexity**	1.15 (1.08-1.26)	0.015	1.26 (1.22-1.56)	0.036
**Ascites (ml)**	1.56 (1.42-1.98)	0.005	1.15 (1.10-1.48)	0.002
**Estimated blood loss (ml)**	1.22 (1.15-1.42)	0.042	1.20 (1.16-1.62)	0.034
**Postop-Chemotherapy (no)**	1.95 (1.24-2.16)	<0.001	2.16 (1.62-2.42)	<0.001
**Lidocaine infusion (yes)**	0.85 (0.78-0.94)	0.026	0.76 (0.62-0.88)	0.032

BMI, Body Mass Index; ASA, American Society of Anesthesiologists score; CCI, Charlson Comorbidity Index; FIGO, Federation International of Gynecology and Obstetrics OS, Overall Survival; DFS, Disease free Survival.

**Table 3:** Multivariable Cox proportional of OS and DFS.

Variables	OS (Before matching)		OS (After matching)		DFS (Before matching)		DFS (After matching)	
	HR (95% CI)	*P*-value	HR (95% CI)	*P*-value	HR (95% CI)	*P*-value	HR (95% CI)	*P*-value
**Lidocaine infusion (yes)**	0.93 (0.82-0.98)	0.026	0.86 (0.62-0.98)	0.038	0.80 (0.62-0.92)	0.019	0.73 (0.62-0.88)	0.046
**Tumor differentiation (poor)**	1.36 (1.02-1.78)	0.011	1.26 (1.22-1.73)	<0.001	1.76 (1.62-1.88)	0.021	1.63 (1.142-1.78)	0.035
**Residual disease (>1cm) **	1.28 (1.23-1.58)	<0.001	1.21 (1.02-1.48)	0.029	1.83 (1.62-1.98)	0.026	1.66 (1.22-1.53)	<0.001
**Postop-Chemotherapy (no)**	1.56 (1.41-1.62)	<0.001	1.16 (1.12-1.43)	0.016	1.93 (1.32-2.28)	<0.001	1.76 (1.12-1.83)	<0.001

OS, Overall Survival; DFS, Disease free Survival.

Moreover, DFS rates at 3 and 5 years after surgery differed between patients in the lidocaine group and those in the non-lidocaine infusion group [3-year DFS: 42.5%, vs. 34.6%, P<0.001, and 5-year DFS: 32.6% vs. 21.3%, P=0.011, respectively, **Figure 1B**]. Univariate Cox regression analysis showed that age, ASA score, CCI, tumour differentiation, FIGO stage, residual disease, surgical complexity, ascites, intraoperative blood loss, postoperative chemotherapy, and lidocaine infusion were associated with DFS (**Table 2**). In the multivariate Cox proportional hazards model before propensity score matching, poor tumour differentiation (HR: 1.36, 95% CI: 1.02, 1.78, P=0.011), residual disease (HR: 1.28, 95%CI: 1.23, 1.58, P<0.001), and no adjuvant chemotherapy (HR: 1.56, 95% CI: 1.41, 1.62, P<0.001) were independent factors of unfavourable DFS (**Table 3**). Intravenous infusions of lidocaine were associated with prolonged DFS (HR: 0.93, 95% CI: 0.82, 0.98, P=0.026), and the association between lidocaine infusion and OS remained statistically significant after propensity score matching (HR: 0.86, 95% CI: 0.62, 0.98, P=0.038). The following covariates were associated with worse DFS: tumour differentiation (HR: 1.26, 95% CI: 1.22, 1.73, P=0.011), residual disease (HR: 1.21, 95%CI: 1.02, 1.48, P=0.029), and adjuvant chemotherapy (HR: 1.16, 95% CI: 1.12, 1.43, P=0.016) (**Table 3**).

### Secondary Outcomes

**Figure 2:**
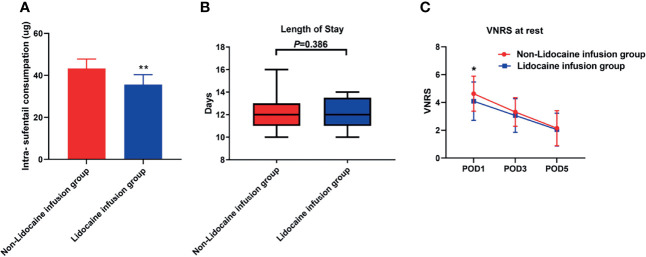
**(A)** Intraoperative sufentanil consumption between groups. **(B)** Length of postoperative hospital stay between groups. **(C)** The verbal numeric rating scale at rest between groups. *P < 0.05, **P < 0.001.

Intraoperative sufentanil consumption was significantly lower in the lidocaine group (Mean (standard deviation, SD),35.6 μg ± 4.8 μg *vs.*43.2 μg ± 4.6 μg, P<0.001, **Figure 2A**) than in the non-lidocaine group. Also, the average VNRS score after surgery on postoperative day 1 in lidocaine infusion group was lower compared with non-lidocaine infusion group. (4.0 ± 1.3 *vs.* 4.7 ± 1.1, P<0.001, **Figure 2C**). In terms of length of hospital stay, the median duration (interquartile) in the non-lidocaine group was 12.4 days [10.0, 13.7], whereas the mean LOS was 12.0 days in the lidocaine group [10.0, 13.1] (P=0.386, **Figure 2B**).

### SCN9A as a Predictor of Ovarian Cancer Survival

**Figure 3:**
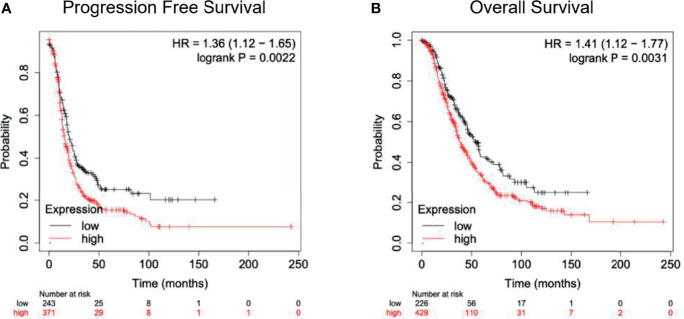
**(A)** Progression-free survival curves from the date of treatment according to SCN9A expression. **(B)** Overall survival curves from the date of treatment according to SCN9A expression.

For mRNA SCN9A expression analysis, we included women with all types of ovarian cancer (stages 1-4) who might have received primary debulking surgery and adjuvant chemotherapies. The mRNA expression analysis demonstrated that *SCN9A* is expressed in ovarian cancer tissues, though at slightly lower levels than in controls (**Figure 3A** and **Table 4**). Kaplan-Meier curves also indicated significantly shorter progression-free survival and OS among women with tumours expressing higher than the median *SCN9A* mRNA level (**Figure 3B**).

**Table 4:** mRNA expression of SCN9A in normal versus ovarian cancer tissue.

Gene	Tissue	Min	Q1	Median	Q3	Max	P value
*SCN9A*	Normal (n=5)	10	43	52	54	57	0.137
	Cancer (n=1,648)	1	15	32	58	706	

Min, minimum; Max, Maximum; Q, Quartile.

## Discussion

The theory that intraoperative administration of lidocaine can improve perioperative outcomes and survival in cancer patients has been the focus of extensive debate and controversy (14). In the present work, we demonstrate that the systemic infusion of lidocaine during ovarian cancer debulking surgery is not only associated with lower opioid use and adequate analgesia but also with improved survival when compared to no intraoperative administration of the local anaesthetic. Our findings deserve several considerations. First, this is the first report suggesting that intraoperative infusion of lidocaine is associated with survival benefits in women with ovarian cancer. Other investigations have been conducted to assess the impact of regional anaesthesia on ovarian cancer progression; however, the results of those studies are mixed (15, 16). In our study, systemic lidocaine was added to epidural analgesia. De Oliveira et al. suggested that adequate and robust intraoperative and postoperative analgesia is associated with better survival in a cohort of women with ovarian cancer (15). Therefore, we speculate that strong modulation of the stress response associated with the use of lidocaine in combination with epidural analgesia may have conferred survival benefits in our cohort of patients. Second, we cannot rule out a direct effect of lidocaine on minimal residual disease. It has been theorized that the long-term effects of anaesthetics in the tumour niche of minimal residual disease are more important in terms of oncological outcomes than is the potential impact in the primary disease. In this regard, lidocaine can modulate several mechanisms involved in cancer cell survival and proliferation as well as enhance the effect of cells involved in immune surveillance. For instance, lidocaine inhibits the invasion and migration of ovarian cancer ES-2 cells, exhibits antiangiogenic effects and stimulates the cytotoxic function of natural killer cells at lower than clinical concentrations (8, 17, 18).

Last, lidocaine is an inhibitor of voltage-gated sodium channels. These channels are expressed at higher levels in ovarian cancer cells with high metastatic potential than in those with low metastatic properties. Our study also showed that the Nav1.7 channel encoded by the gene *SCN9A* may play a role in ovarian cancer cell biology: Patients with high expression of this gene showed worse survival. Therefore, lidocaine might induce apoptosis in minimal residual disease by acting on voltage-gated sodium channels, as indicated in previous studies (19).

Our work has several limitations that are mostly with regard to its retrospective design. Therefore, significant bias and confounding factors related to unknown and unmeasured variables may have influenced our findings, such as postoperative complications and time to adjuvant chemotherapy initiation. We cannot exclude the possibility that the women in the lidocaine group started adjuvant chemotherapy earlier than those in the non-lidocaine group. Additionally, we did not include women who did not receive epidural analgesia. Therefore, it remains unknown whether the associated beneficial effects of lidocaine can be extended to women unable to receive epidural analgesia.

In conclusion, intraoperative intravenous lidocaine infusion during ovarian cancer surgery is associated with a reduction in intraoperative sufentanil consumption, adequate postoperative analgesia and longer PFS and OS. We consider that it is necessary to test our hypothesis under the scientific rigour of a randomized controlled trial.

## Data Availability Statement

The datasets presented in this study can be found in online repositories. The names of the repository/repositories and accession number(s) can be found in the article/supplementary material.

## Ethics Statement

The studies involving human participants were reviewed and approved by the Fudan University. The patients/participants provided their written informed consent to participate in this study.

## Author Contributions

Conception and design: HZ, WC, JC, and CM. Administrative support: HZ, JC, and ZS. Provision of study materials or patients: HZ, JG, and WC. Collection and assembly of data: HZ, JG, JC, and ZS. Data analysis and interpretation: HZ, JG, JC and ZS. Manuscript writing: All authors. Final approval of manuscript: All authors.

## Funding

This research was supported by the National Natural Science Foundation of China (NO. 82102253, 81871591), Clinical Research Plan of SHDC (NO. SHDC2020CR4064), the Shanghai Municipal 2021 “Science and Technology Innovation Action Plan” (NO. 21S31902600), Natural Science Foundation of Shanghai (NO. 21ZR1413400), Shanghai Sailing Program (NO. 21YF1406800), 2019 Fudan University Zhuo-Xue Project (NO. JIF159607).

## Conflict of Interest

The authors declare that the research was conducted in the absence of any commercial or financial relationships that could be construed as a potential conflict of interest.

## Publisher’s Note

All claims expressed in this article are solely those of the authors and do not necessarily represent those of their affiliated organizations, or those of the publisher, the editors and the reviewers. Any product that may be evaluated in this article, or claim that may be made by its manufacturer, is not guaranteed or endorsed by the publisher.
